# Sagittal, rotational and transverse changes with three intraoral distalization force systems: Jones jig, distal jet and first class

**DOI:** 10.4317/jced.57993

**Published:** 2021-05-01

**Authors:** Silvio-Augusto Bellini-Pereira, Aron Aliaga-Del Castillo, Lorena Vilanova, Mayara-Paim Patel, Rachelle-Simões Reis, Roberto-Henrique-da Costa Grec, José-Fernando-Castanha Henriques, Guilherme Janson

**Affiliations:** 1 DDS, MSc, Postgraduate Student. Department of Orthodontics. Bauru Dental School. University of São Paulo, Brazil; 2 DDS, MSc, PhD. Assistant Professor. Department of Orthodontics. University of Guarulhos, São Paulo, Brazil; 3 DDS, MSc, PhD. Department of Orthodontics. Bauru Dental School. University of São Paulo, Brazil; 4 DDS, MSc, PhD. Professor. Department of Orthodontics. Bauru Dental School. University of São Paulo, Brazil; 5 DDS, MSc, PhD. Professor and Head. Department of Orthodontics. Bauru Dental School, University of São Paulo, Brazil

## Abstract

**Background:**

To compare the maxillary dentoalveolar changes of patients treated with three distalization force systems: Jones Jig, Distal Jet and First Class appliances, using digitized models.

**Material and Methods:**

The retrospective sample comprised 118 digitized models of 59 patients with Class II malocclusion divided into three groups: Group 1 consisted of 22 patients treated with the Jones Jig appliance; Group 2 consisted of 20 patients treated with the Distal Jet, and Group 3 comprised 17 patients treated with the First Class appliance. Pretreatment and post-distalization plaster models of all patients were digitized and evaluated with OrthoAnalyzerTM software. The pretreatment and post-distalization variables regarding sagittal, rotational and transverse changes were compared by the One-way Analysis of Variance (ANOVA) and Kruskal-Wallis tests, depending on normality.

**Results:**

All appliances presented similar amounts of distalization. The Distal Jet appliance promoted significantly smaller mesial displacement of premolars and greater expansion of posterior teeth. The First Class presented the smallest rotation of the maxillary molars and treatment time.

**Conclusions:**

The distalizers were effective in correcting Class II molar relationship, however, a palatal force seems to provide fewer undesirable effects. Additionally, the degree of rotation and expansion was associated with the side of force application.

** Key words:**Malocclusion, Angle Class II, Orthodontics, Corrective, Distalizers.

## Introduction

Intraoral distalizers require minimum patient cooperation, becoming a common alternative to correct Class II molar relationship. These devices include magnets (1), nickel-titanium (NiTi) springs (2), Pendulum (3,4), Jones Jig (5,6), First Class (7), Distal Jet (8), among others.

Even though effective to obtain maxillary molar distalization, the use of conventionally anchored distalizers is controversial since it is related do undesirable effects, such as anterior anchorage loss and premolars mesialization (9). The design of these appliances may be related to the amount of distalization promoted and these possible undesirable effects. Therefore, different changes could be expected with the Jones Jig, that applies a buccal distalization force, the Distal Jet that uses a palatal distalization force, and the First Class which applies buccal and palatal forces.

Previous studies investigated the dentoskeletal effects of distalizers. However, most of them were performed using cephalometric variables (6,8,10,11). Thus, there is still a deficiency in the evaluation of some clinically relevant aspects, especially regarding rotation and transverse changes after the use of distalizing appliances (12). Knowledge of these dentoalveolar effects and the extent of their possible side effects could influence decision-making during treatment planning.

Therefore, the purpose of this study was to compare the maxillary sagittal, rotational and transverse changes of patients treated with three different distalization force systems: Jones Jig, Distal Jet and First Class appliances, using digitized models.

## Material and Methods

This retrospective study was approved by the Ethics in Research Committee of Bauru Dental School, University of São Paulo (Protocol number: 71639017.0.000.5417).”

Sample size calculation was based on an alpha significance level of 5% and a beta of 20%, to detect a mean difference of 2.00 mm, with a standard deviation of 1.40 mm in the sagittal displacement of the maxillary first molar, as reported in a previous study (13). A minimum of 11 patients were required in each group based on the sample size calculation.

-Sample characteristics

The sample comprised 118 digitized models of 59 patients (23 male, 36 female) divided into 3 groups, treated at the Department of Orthodontics, Bauru Dental School, University of São Paulo, Brazil.”. The inclusion criteria were based on the following characteristics: 1. Presence of Class II malocclusion; 2. No severe skeletal discrepancies; 3. No severe maxillary and mandibular crowding; 4. No crossbite; 5. Absence of previous orthodontic treatment.

**Figure 1:**
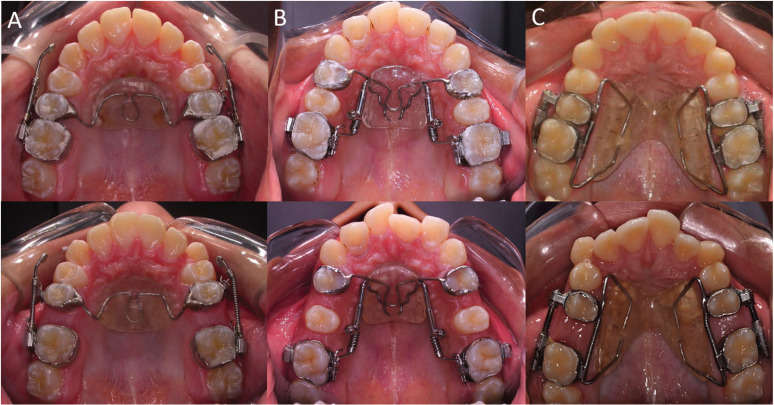
Pre- and post-distalization intraoral photographs: A) Jones Jig appliance; B) Distal Jet appliance; C) First Class appliance.

Group 1 consisted of 22 patients (10 male, 12 female) with a mean initial age of 13.03 years (± 1.05 years), treated with the Jones Jig appliance (Fig. 1A). In order to exert a continuous force (120g) to the molars, the appliance was built with a nickel-titanium (NiTi) coil spring (American Orthodontics, Sheboygan, Wisc), activated 5mm every 4 weeks. The anchorage unit used was a Nance button attached to the second premolars (6).

Group 2 comprised 20 patients (6 male, 14 female) with a mean initial age of 12.25 years (± 1.38), treated with the Distal Jet appliance (Fig. 1B). The open-coils springs of the appliance were selected to exert 240g of force in patients with the second molars erupted and 180g in those with absence of these teeth. The appliance was reactivated once a month, and after distalization the Nance button was converted to a Nance holding arch (8).

Group 3 consisted of 17 patients (6 male, 11 female) with a mean initial age of 13.14 years (± 1.41), treated with the First Class appliance (Fig. 1C). The appliance delivered forces from the buccal and palatal sides, with activation screws and NiTi coil springs (10mm long) respectively. A modified Nance butterfly-shaped button was used as the anchorage unit, and the appliance was activated rotating the screws in a counterclockwise direction once a day (7,10).

In all three groups, distalization was performed aiming overcorrection until a super-Class I relationship was obtained (14). The mean distalization time was 0.86 years (± 0.31); 0.95 years (± 0.31); and 0.69 years (± 0.21), for the Jones Jig, Distal Jet and First Class appliances, respectively.

-Digitized Model Analyses

Plaster models before (T0) and after molar distalization (T1) from all patients were submitted to 3D surface laser scanning by the 3Shape R700 scanner (3Shape, Copenhagen, Denmark). The scanner generated three-dimensional images of all plaster models (n=118). Therefore, pre- and post-distalization scans were analyzed with the OrthoAnalyzerTM software (3Shape Ltd, Copenhagen, Denmark), following an adapted method previously described (15).

**Figure 2:**
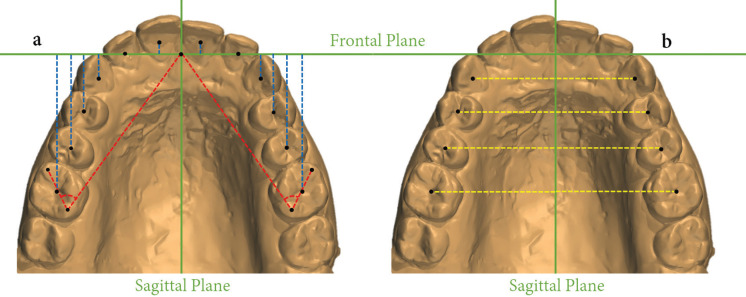
Measurements performed on the digitized models. Centroid points (black) and reference planes (green). a) Sagittal (blue) and rotational (red) measurements. b) Transverse measurements (yellow).

A frontal plane, perpendicular to the sagittal plane, and passing through the most anterior point of the incisive papilla was constructed by the software on the digitized models to determine the sagittal changes of the incisors, canines, premolars and molars (15) (Fig. 2A). Then, perpendicular lines from the centroid point of the teeth to the frontal plane were drawn. Positive values indicated distal teeth displacement, while negative values indicated mesial displacement.

To quantify the degree of molar rotation, two lines were constructed. One, passing through the most anterior point of the incisive papilla, to the tip of the distopalatal cusp of the maxillary first molar, and another line connecting the tips of the mesiobuccal and distopalatal cusps of the same molar (Fig. 2A). The angle formed by the intersection of these two lines was used (15). An increase of the angle indicated distal rotation of the molar during treatment and a decrease, mesial rotation.

To measure the amount of transversal changes, the sagittal plane was used (15). Thus, the distance from this plane to the canines, premolars and molars centroids was measured (Fig. 2B). Positive values represented buccal displacement (expansion), and negative values, palatal displacement (constriction).

All these measurements were performed on the digitized models before (T0) and after distalization (T1), and the treatment changes (T1-T0) were compared.

-Error Study

After a month interval from the first measurements, 20 randomly selected models were re-digitized and remeasured by the same examiner (S.A.B.P.). The random errors were estimated according to Dahlberg’s formula (16), while the systematic errors were calculated with dependent t tests, at *P*<0.05 (17).

-Statistical Analysis

Normal distribution was evaluated by Shapiro Wilk tests in the three groups, in both treatment stages and also for the treatment changes (T0 and T1) for all teeth.

Intergroup comparability regarding sex, Class II molar relationship severity distributions and presence or absence of second molars (7s) were analyzed with Chi-square tests. Pre- and posttreatment ages and treatment time intergroup comparability were evaluated with One-way Analysis of Variance (ANOVA), followed by Tukey tests.

Intergroup pretreatment stage and treatment changes (T1-T0) were compared with ANOVA, followed by Tukey tests for the variables with normal distribution, and with Kruskal Wallis tests for those without normal distribution.

## Results

The random errors ranged from 0.10 mm to 0.42 mm (sagittal displacement of teeth 24 and 22 respectively) (18), and from 0.89° to 0.92° (rotation of teeth 16 and 26), considered inside the accepTable limits for clinical implication (19). No systematic errors were found.

The groups were comparable regarding sex, Class II malocclusion severity distributions, presence or absence of second molars (7s), pre- and posttreatment ages (Table 1). However, the First Class group presented a significantly smaller treatment time when compared to the Distal Jet group.

**Table 1:**
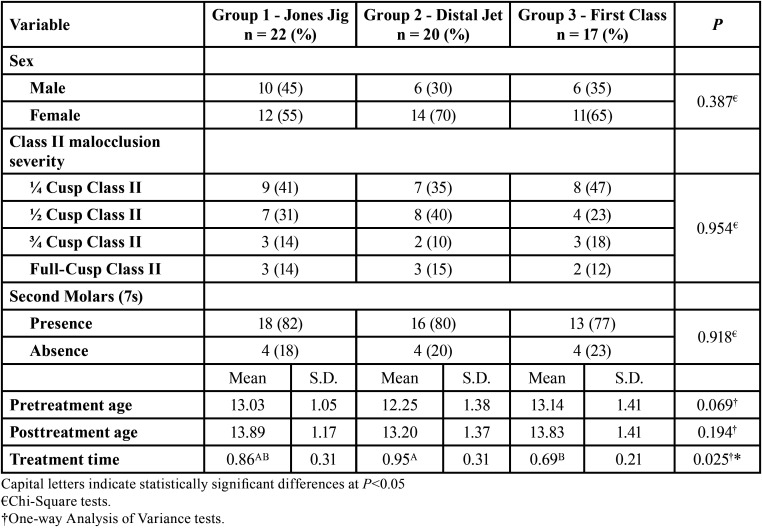
Intergroup comparison of sex, Class II malocclusion severity distributions, presence or absence of second molars (7s), pre- and posttreatment ages, and treatment times.

Pretreatment intergroup comparison showed the left first molar and second premolar significantly more palatally located in the Distal Jet group when compared to the First Class group (Table 2).

**Table 2:**
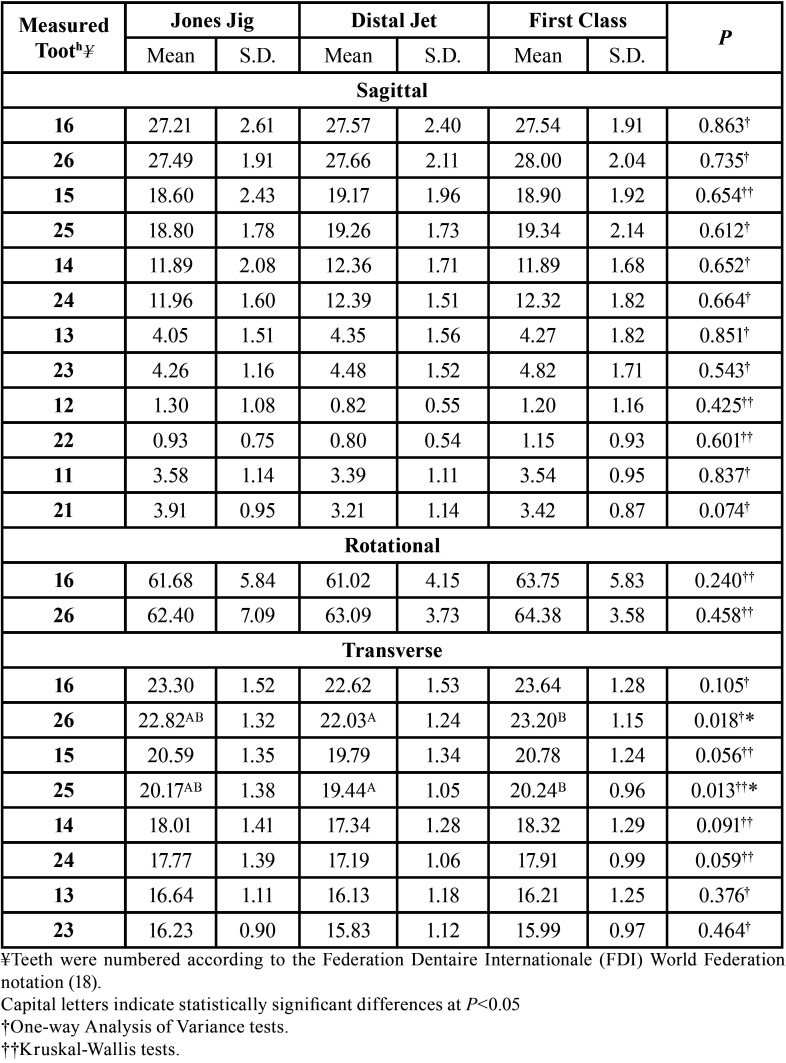
Pretreatment intergroup comparison (One-way Analysis of Variance and Kruskal-Wallis tests).

During treatment, the second premolars in the Distal jet group moved distally, while in the Jones Jig and First Class groups mesial displacement was observed, therefore, demonstrating significant differences (Table 3). The Jones Jig group presented significantly greater mesial displacement of the first premolars when compared with the Distal Jet group.

**Table 3:**
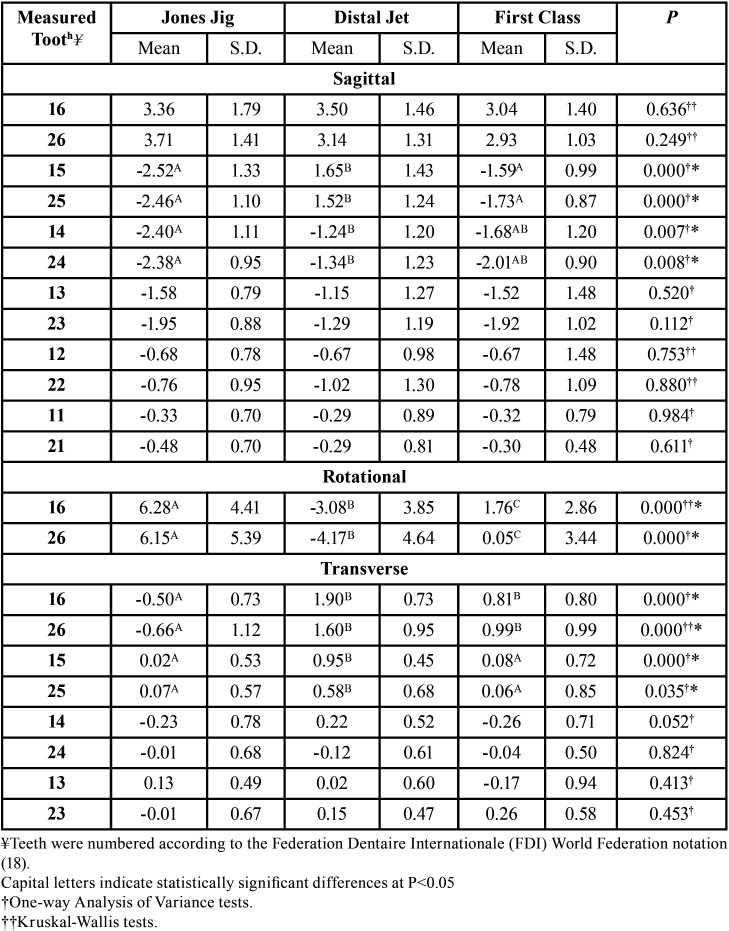
Intergroup treatment changes comparison (One-way Analysis of Variance and Kruskal-Wallis tests).

The Jones Jig and First Class groups presented distal rotation, while the Distal Jet group showed mesial rotation, therefore with significant differences (Table 3). Significantly different behavior between the groups was also observed in the first molars transversal displacement, with the Jones Jig group resulting in palatal displacement and the other two groups presenting buccal displacement. Moreover, the second premolars in the Distal Jet group showed significantly greater buccal displacement than the Jones Jig and First Class groups.

## Discussion

Previous studies evaluated the dentoskeletal effects of distalizers by means of cephalometric analysis after distalization (11,20) and after orthodontic treatment (21). However, few studies (12,22) evaluated the rotational and transversal dental changes induced by these appliances, especially using distalizers with different sites of force application. Therefore, this study compared three distalization systems, with different characteristics, using digitized models to evaluate the dentoalveolar changes during distalization.

-Sample Characteristics

It could be considered that the retrospective design of this study may give rise to selection bias and other biases (23). However, the presence of this inherent methodological limitation should be overcome by the great intergroup comparability. The groups were quite similar in terms of sex, Class II malocclusion severity distributions, presence, and absence of second molars, pre- and posttreatment ages (Table 1).

One may argue that due to the amount of variables and comparisons between groups, Bonferroni corrections (24) should have been performed. However, the probability of detecting slight significant differences would be smaller if the correction was performed, and these small differences could be important in this comparison.

Furthermore, the treatment time of the groups was inside the limits suggested by previous studies (9,25). Additionally, the shorter treatment time presented by the First Class appliance was similar to previous reports (7,26).

At the pretreatment stage, the majority of dental characteristics was also comparable between the groups (Table 2). The first molar and second premolar significantly more palatally located in the Distal Jet group might not have interfered in the results, since this difference could be considered without clinical significance and did not alter the performance of the distalizers.

-Sagittal Changes

The major objective of using intraoral distalizing appliances is to correct molar relationship by distalizing the molars until a super-Class I is achieved. In this study, all the appliances tested were capable to perform molar distalization effectively with amounts ranging from 2.93 to 3.71 mm (Table 3). These findings corroborate with other studies that presented similar amounts of distalization using these appliances (26,27).

Effectiveness of these appliances is controversial since their use is commonly associated with undesirable effects such as premolars mesial displacement and incisors protrusion (9,28). However, in the case of the second premolars, the groups behaved differently, since the second premolars in the Distal Jet group followed the molar distal movement (Table 3). The anchorage unit of the Distal Jet appliance is supported on the first premolars, therefore, allowing the second premolars to drift distally under the pulling effect of the transseptal fibers (22,28). Nonetheless, as expected, the first premolars from all groups presented mesial displacement. The Jones Jig group presented the greater displacement, similar to other studies (5,29).

The anterior teeth from all groups presented mild protrusion (Table 3). This anchorage loss can be reflected in a clinically significant increase in the overjet (26). These findings are suggested in most studies with intraoral distalizing appliances conventionally anchored (5,11,20,26,28). It could be possible that the anchorage unit of the appliances was insufficient to counteract the reciprocal distalization force, and the use of skeletal anchorage is the only possibility to reduce or even prevent anterior anchorage loss (9,29).

-Rotational and Transverse Changes

In the case of the Jones Jig, the force applied from the buccal side promoted distal rotation (29,30) (Table 3). Thus, a force from the palatal side promoted mesial rotation, which was the case for the Distal Jet appliance (28). Furthermore, the First Class group showed distalization without significant rotational effects since the force was applied from both sides. It is reasonable to state that molar rotation is directly affected by the side of force application, whether from the buccal, palatal or both sides.

A recent study compared the buccally acting Karad’s Integrated Distalizing System (KIDS) with the palatally acting Frog appliance (12). The first, promoted maxillary molar distal rotation ranging from 5.5° to 6.3°, and the Frog appliance showed mesial rotation ranging from 4.4° to 5.9°. Our findings are consistent with those, and showed similar amounts of rotation with a force applied from the buccal (Jones Jig) and palatal (Distal Jet) sides (Table 3). Another study (22) obtained greater mesial rotation, ranging from 7.88° to 8.35° with a skeletonized Distal Jet appliance, suggesting that skeletal anchorage might have small impact on molar rotation.

These findings may be extrapolated even when distalization is associated with skeletal anchorage. The orthodontist should consider the initial molar status and correctly plan the side of force application.

Regarding the transversal aspect, the significant changes during distalization were concentrated on the molars and second premolars (Table 3). The Jones Jig group showed significant palatal displacement of the molars. The moment of force produced by the coil spring caused distal rotation and this could probably reflect in a tendency of a posterior crossbite in some teeth (29). Nevertheless, these transversal changes on the molars did not exceed 1 mm, and may not have clinical significance. On the other hand, the Distal Jet and First Class groups presented buccal displacement of the molars, representing mild expansion, as previously demonstrated (22,26,28).

The Distal Jet group showed significantly greater buccal displacement of the second premolars when compared to the other groups (Table 3). This was already expected, since the anchorage unit is supported by these teeth in the Jones Jig and First Class appliances, resulting in no transversal changes. Differently, the second premolars in the Distal Jet groups were able to follow molar distalization and expansion (22,28).

Orthodontists should understand the dentoalveolar effects promoted by distalizers and their undesirable effects, to comprehend the benefits of associating these appliances with skeletal anchorage.

Selection of the appliance design must consider cost-effectiveness, fewer undesirable effects, and the patients’ assumptions. The appliances compared in this study were able to achieve maxillary molar distalization. However, after their use, orthodontic mechanics should be applied to correct the undesirable effects inherent by the use of conventional anchorage.

## Conclusions

- Correction of the Class II molar relationship was effectively obtained with the appliances tested. Similar amounts of distalization were promoted with some degree of undesirable effects.

- The Distal Jet appliance promoted smaller mesial displacement of premolars and greater expansion of posterior teeth.

- The First Class promoted the smallest rotation of maxillary molars and had the smallest treatment time.

- The degree of molar rotation and expansion was associated with the side of force application.

## References

[1] Gianelly AA, Vaitas AS, Thomas WM (1989). The use of magnets to move molars distally. American Journal of Orthodontics and Dentofacial Orthopedics.

[2] Gianelly AA, Bednar J, Dietz VS (1991). Japanese NiTi coils used to move molars distally. American Journal of Orthodontics and Dentofacial Orthopedics.

[3] Angelieri F, de Almeida RR, de Almeida MR, Fuziy A (2006). Dentoalveolar and skeletal changes associated with the pendulum appliance followed by fixed orthodontic treatment. American Journal of Orthodontics and Dentofacial Orthopedics.

[4] Chaques-Asensi J, Kalra V (2001). Effects of the pendulum appliance on the dentofacial complex. Journal of Clinical Orthodontics.

[5] Brickman CD, Sinha PK, Nanda RS (2000). Evaluation of the Jones jig appliance for distal molar movement. American Journal of Orthodontics and Dentofacial Orthopedics.

[6] Jones RD, White JM (1992). Rapid Class II molar correction with an open-coil jig. Journal of Clinical Orthodontics.

[7] Fortini A, Lupoli M, Giuntoli F, Franchi L (2004). Dentoskeletal effects induced by rapid molar distalization with the first class appliance. American Journal of Orthodontics and Dentofacial Orthopedics.

[8] Carano A, Testa M, Siciliani G (1996). The Distal Jet for uprighting lower molars. Journal of Clinical Orthodontics.

[9] Grec RH, Janson G, Branco NC, Moura-Grec PG, Patel MP, Castanha Henriques JF (2013). Intraoral distalizer effects with conventional and skeletal anchorage: a meta-analysis. American Journal of Orthodontics and Dentofacial Orthopedics.

[10] Fortini A, Lupoli M, Parri M (1999). The First Class Appliance for rapid molar distalization. Journal of Clinical Orthodontics.

[11] Patel MP, Janson G, Henriques JF, de Almeida RR, de Freitas MR, Pinzan A (2009). Comparative distalization effects of Jones jig and pendulum appliances. American Journal of Orthodontics and Dentofacial Orthopedics.

[12] Uzuner FD, Kaygisiz E, Unver F, Tortop T (2016). Comparison of transverse dental changes induced by the palatally applied Frog appliance and buccally applied Karad's integrated distalizing system. Korean Journal of Orthodontics.

[13] Ali D, Mohammed H, Koo SH, Kang KH, Kim SC (2016). Three-dimensional evaluation of tooth movement in Class II malocclusions treated without extraction by orthodontic mini-implant anchorage. Korean Journal of Orthodontics.

[14] Ferguson DJ, Carano A, Bowman SJ, Davis EC, Gutierrez Vega ME, Lee SH (2005). A comparison of two maxillary molar distalizing appliances with the distal jet. World Journal of Orthodontics.

[15] Nalcaci R, Kocoglu-Altan AB, Bicakci AA, Ozturk F, Babacan H (2015). A reliable method for evaluating upper molar distalization: Superimposition of three-dimensional digital models. Korean Journal of Orthodontics.

[16] Dahlberg G (1940). Statistical methods for medical and biological students. Br Med J.

[17] Houston WJ (1983). The analysis of errors in orthodontic measurements. American Journal of Orthodontics.

[18] Staff E (2001). Federation Dentaire Internationale (FDI) tooth-numbering system. American Journal of Orthodontics and Dentofacial Orthopedics.

[19] Mavropoulos A, Karamouzos A, Kiliaridis S, Papadopoulos MA (2005). Efficiency of noncompliance simultaneous first and second upper molar distalization: a three-dimensional tooth movement analysis. Angle Orthodontist.

[20] Chiu PP, McNamara JA Jr, Franchi L (2005). A comparison of two intraoral molar distalization appliances: distal jet versus pendulum. American Journal of Orthodontics and Dentofacial Orthopedics.

[21] Patel MP, Henriques JFC, Freitas KMdS, Grec RHdC (2014). Cephalometric effects of the Jones Jig appliance followed by fixed appliances in Class II malocclusion treatment. Dental Press Journal of Orthodontics.

[22] Kinzinger GSM, Gülden N, Yildizhan F, Diedrich PR (2009). Efficiency of a skeletonized distal jet appliance supported by miniscrew anchorage for noncompliance maxillary molar distalization. American Journal of Orthodontics and Dentofacial Orthopedics.

[23] Dalziel K, Round A, Stein K, Garside R, Castelnuovo E, Payne L (2005). Do the findings of case series studies vary significantly according to methodological characteristics?. Health Technol Assess.

[24] Armstrong RA (2014). When to use the Bonferroni correction. Ophthalmic Physiological Optics.

[25] Bellini-Pereira SA, Pupulim DC, Aliaga-Del Castillo A, Henriques JFC, Janson G (2019). Time of maxillary molar distalization with non-compliance intraoral distalizing appliances: a meta-analysis. European Journal of Orthodontics.

[26] Papadopoulos MA, Melkos AB, Athanasiou AE (2010). Noncompliance maxillary molar distalization with the first class appliance: a randomized controlled trial. American Journal of Orthodontics and Dentofacial Orthopedics.

[27] Cozzani M, Pasini M, Zallio F, Ritucci R, Mutinelli S, Mazzotta L (2014). Comparison of maxillary molar distalization with an implant-supported distal jet and a traditional tooth-supported distal jet appliance. International Journal of Dentistry.

[28] Bowman S (2016). Upper-Molar Distalization and the Distal Jet. J Clin Orthod.

[29] Runge ME, Martin JT, Bukai F (1999). Analysis of rapid maxillary molar distal movement without patient cooperation. Am J Orthod Dentofacial Orthop.

[30] Paul L, O'brien K, Mandall N (2002). Upper removable appliance or Jones Jig for distalizing first molars? A randomized clinical trial. Orthodontics & Craniofacial Research.

